# The complete chloroplast genome sequence of *Sibiraea angustata*, a traditional Chinese medicine in Sichuan Province, China

**DOI:** 10.1080/23802359.2020.1866460

**Published:** 2021-02-04

**Authors:** Jiayong Li, Zhihang Zhuo, Danping Xu, Hongjun Yang, Tianhui Zhu

**Affiliations:** a College of Forestry, Sichuan Agricultural University, Chengdu, Sichuan, China; b College of Life Science, China West Normal University, Nanchong, Sichuan, China; c College of Forestry, Hainan University, Haikou, Hainan, China

**Keywords:** *Sibiraea angustata* Illumina NovaSeq chloroplast genome phylogenetic analysis

## Abstract

The complete chloroplast genome of *Sibiraea angustata* was assembled and subjected to phylogenetic analysis. The chloroplast genome of *S. angustata* was 155,869 bp in length, containing a large single-copy region (84,343 bp), a small single-copy region (18,820 bp), and two inverted repeat regions (26,353 bp). The overall GC content of *S. angustata* chloroplast genome was 36.80%. The chloroplast genome of *S. angustata* contained 127 unique genes, including 83 protein-coding genes, 36 tRNA genes and eight rRNA genes. Phylogenetic analysis revealed that *S. angustata* was related to *Malus ioensis*, *Malus florentina* and *Malus trilobata*.

*Sibiraea angustata* (Rehd.) Hand.-Mazz. is a typical plant in the Qinghai-Tibet Plateau, mainly distributed in the shrub of 3000–4000 m in Western China (Wu 1998). The twigs and fresh leaves of *S. angustata* are used as traditional Chinese medicine to treat stomach discomfort and indigestion (Liu et al. 2020). To make better use of *S. angustata*, the complete chloroplast genome of *S. angustata* was assembled and subjected to phylogenetic analysis.

Fresh leaves of *S. angustata* were sampled from Ruoergai County, Aba State, Sichuan Province, China (N33°37′45.55″, E102°36′20.14″). The specimen was deposited in the Laboratory of Forest Pathology, College of Forestry, Sichuan Agricultural University (Voucher No. SAHM-202005177). Total genomic DNA was extracted according to the mCTAB protocol (Li et al. 2013). The genome sequencing was conducted using Illumina NovaSeq platform by Shanghai Personal Biotechnology Co. Ltd, China. The chloroplast genome of *S. angustata* was assembled with SPAdes v3.6.0 software (Bankevich et al. 2012) and annotated with Plann (Huang and Cronk 2015). The complete chloroplast genome sequence of *S. angustata* has been submitted to the GenBank (MW123094).

The chloroplast genome of *S. angustata* was 155,869 bp in length, containing a large single-copy (LSC) region (84,343 bp), a small single-copy (SSC) region (18,820 bp), and two inverted repeat (IR) regions (26,353 bp). The overall GC content of *S. angustata* chloroplast genome was 36.80%, and in the SSC, LSC, and IR regions were 30.64%, 34.61%, and 42.51%, respectively. The chloroplast genome contained 127 complete genes, including 8 rRNA genes, 36 tRNA genes, and 83 protein-coding genes.

To evaluate the phylogenetic relationships of *S. angustata* within Rosaceae, the chloroplast genome sequence of *S. angustata* and those of 12 other Rosaceae species were aligned by MAFFT v7 (Katoh and Standley 2013), and the phylogenetic tree was constructed with MEGA X using neighbour-joining analysis (Kumar et al. 2018). The phylogenetic tree revealed that *S. angustata* was most related with *Malus ioensis*, *Malus florentina* and *Malus trilobata* (Figure 1).

**Figure 1.**
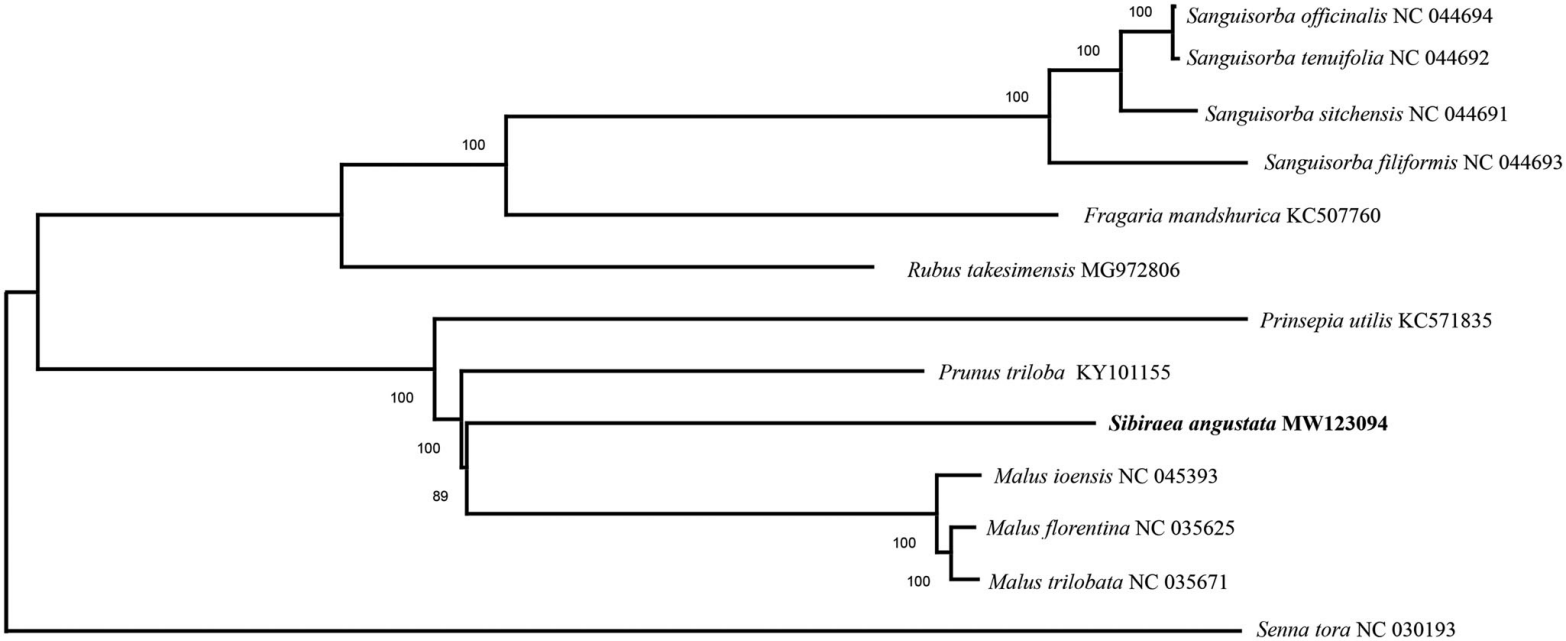
The phylogenetic relationship of 13 species within the Rosaceae species based on neighbour-joining analysis of complete chloroplast genomes. *Senna tora* was served as the out-group.

## Date availability statements

The genome sequence data that support the findings of this study are openly available in GenBank of NCBI at (https://www.ncbi.nlm.nih.gov/) under the accession number MW123094. The associated BioProject, SRA, and Bio-Sample numbers are PRJNA669189, SUB8684720, and SAMN16457089 respectively.
